# 

**Published:** 1885-07-20

**Authors:** 


### EDITORIALS AND MISCELLANEOUS.

☞ **Notice.**--- Subscribers in arrears are requested to remit their dues at once. Please take due notice and govern yourselves accordingly.

### EDITORIAL NOTICES.

SOUTHERN MEDICAL COLLEGE.—See the advertisement of this School in this issue.

WM. WARNER & Co. have received the first premium at the World's Exposition, New Orleans, for great uniformity and solubility for their sugar-coated pills. This is the ninth World's Fair prize which attests to their excellence.

DR. W. G. BULLOCH, of Savannah, Ga., died on June 24, 1885, aged seventy-five years. Dr. Bulloch was a graduate of Yale College and of the University of Pennsylvania, Medical Department. He was an honored and prominent physician, and his memory will be cherished by numerous friends and relatives in Georgia.

TYPHOID EPIDEMIC.—It is claimed that the terrible epidemic of Typhoid Fever that recently prevailed at Plymouth, Pa., has been traced to contamination of the water supply consequent upon dejecta from a single patient thrown upon the snow within a few feet of one of the sources of supply.

"DON'T VIEW ME WITH A RABBIT'S EYE."—The old saying in regard to "casting sheep's eyes" must now be changed, as it is authoritatively announced that Dr. Chilret, a famous occulist of France, reported in May last to the Academie de Medicine that, having removed a diseased eye from a young girl, he substituted a rabbit's eye in its place, and that, though two weeks had elapsed since the operation, "the eye held well," and there was every prospect of its proving a complete success !

PEACOCK'S FUCUS MARINA, AND PEACOCK'S BROMIDES.—Samples of these preparations have been kindly furnished us for trial, and we must say that we are very much pleased with them. See their advertisement in this journal.

THE MISSISSIPPI VALLEY MEDICAL SOCIETY (formerly Tri-State) will meet in Evansville, Ind., Tuesday, September 8, at 10 o'clock a. m., and continue through the 9th and 10th. The following are the officers of the Society. We note that Dr. Arch. Dixon, of Henderson, Ky., one of the able contributors to this journal, is in charge of the important Section on Practice and Pathology—a good appointment truly :

President, Dr. F. W. Beard, Vincennes, Ind.
First Vice-President, Dr. A. B. Miller, Missouri.
Second Vice-President, Dr. J. A. Sutcliffe, Indiana.
Third Vice-President, Dr. E. H. Luckett, Kentucky.
Secretary, Dr. G. W. Burton, Mitchell, Ind.
Assistant Secretary, Dr. R. J. B. Wright, Illinois.

### *RECEIPTED.*

1885, July—I. E. Martin, W. E. Courser, G. L. Mills, Samuel Pool, R. M. Savage.
1883—P. M. Catching, A. J. Burkett (to July 86).
1884—*Wyly Mills, James Dobbin, Rob't Hance, T. L. Munhall, R. C. Wickliff, T. J. Miller, Thomas Jilds, Moses Worth.*

